# Real Steps or Not: Auto-Walker Detection in Move-to-Earn Applications

**DOI:** 10.3390/s25041002

**Published:** 2025-02-07

**Authors:** Sunwoo Lee

**Affiliations:** Department of Information Security, Seoul Women’s University, 621, Hwarang-ro, Nowon-gu, Seoul 01797, Republic of Korea; sun.lee@swu.ac.kr

**Keywords:** fraud detection, M2E, gait, MEMS sensor, AI, signal processing

## Abstract

In recent times, the emergence of Move-to-Earn (M2E) applications has revolutionized the intersection of digital innovation and physical wellness. Unlike their predecessors in the Play-to-Earn (P2E) domain, M2E apps incentivize physical activity, offering rewards for real-world movement such as walking or running. This shift aligns with a growing global focus on health consciousness that is propelled by the widespread adoption of smartphones and an increased awareness of the benefits of maintaining an active lifestyle. However, the rising popularity of these platforms has also brought about new problematic activities, with some users exploiting additional automated devices to simulate physical activity and claim rewards. In response, we propose an AI-based method aimed at distinguishing genuine user engagement from artificially generated auto-walker activity to ensure the integrity of reward distributions in M2E platforms. To demonstrate the generalizability of our model, we use a total of six open gait datasets and auto-walker datasets of automatic walking devices measured with various smartphones. Under unbiased and transparent evaluation, our model shows its ability to effectively discriminate auto-walker and genuine gait data not only on the seen datasets but also on the unseen datasets; it attained an F1-score of 0.997 on the auto-walker datasets and an F1-score of 1.000 on the genuine datasets.

## 1. Introduction

In recent times, there has been a significant surge in popularity of reward-based applications, particularly against the backdrop of the economic climate defined by the “three highs”: elevated inflation, interest rates, and exchange rates. This period has compelled consumers to adopt a more frugal mindset and actively seek avenues to reduce personal expenses by capitalizing on savings opportunities, however minor. Amid this economic landscape, reward apps have emerged as a promising avenue, as they offer users the opportunity to earn monetary rewards for completing simple tasks through the app interface. These tasks range from watching advertisements and answering quizzes to achieving specific step counts, effectively monetizing everyday activities. Among the myriad of reward-based applications, Move-to-Earn (M2E) apps have become distinguished. Evolving from the Play-to-Earn (P2E) model, which primarily rewarded users within gaming environments, M2E apps shift the focus toward promoting healthier lifestyles. By incentivizing physical activities such as walking or running, these apps aim to reward users who incorporate more active routines into their daily lives. A report by Grand View Research, Inc. projects that the global fitness apps market encompassing M2E applications will reach USD 1.8 billion by 2030, with an expected compound annual growth rate (CAGR) of 18.3% during the forecast period [1]. This growth is propelled by the widespread adoption of smartphones and a growing consciousness about the importance of maintaining a healthy lifestyle. As the prioritization of well-being becomes more prevalent in society, we can expect that the appeal and uptake of M2E apps will rise significantly as well, posing as integral tools that foster a culture of holistic health and wellness. A notable advantage of these app-based reward systems is the flexibility they offer users to simultaneously engage with multiple platforms. For instance, a user can amplify potential rewards by installing several apps that reward similar activities, such as achieving a specific step count, effectively maximizing their earnings from the platforms.

However, a concerning trend has grown in parallel with the rising popularity of reward apps: the emergence of users seeking to exploit these platforms for financial gain through deceptive means. One prevalent method involves the use of automatic walking devices, or “auto-walkers”, designed to simulate steps without actual physical movements. While companies operating these apps expect rewards to be distributed based on genuine user engagement and physical exertion, some users utilize automatic walking devices to claim rewards for performing the action. These practices enable users to accumulate rewards at an accelerated rate—one that is well beyond what companies intended.

This paper addresses the challenge of distinguishing genuine human activities from auto-walker-generated movements in Move-to-Earn (M2E) applications. We propose an AI-driven approach that analyzes gait signals—capturing differences in movement patterns between actual human walking and artificially generated motion from automatic walking devices as detected by smartphone sensors. While most existing studies on gait analysis have focused on human activity recognition (HAR) [2,3,4,5] or disease diagnosis [6,7,8], anomaly detection in digital platforms has primarily been applied to financial fraud detection, identifying suspicious transactions and behavioral anomalies [9]. However, these methods are not designed to detect motion-based fraud in M2E applications. Our work is the first to specifically address this issue, introducing a robust fraud detection mechanism that ensures the integrity of reward-based platforms. By accurately identifying fraudulent activity, our method helps prevent exploitation, ensuring that rewards are distributed fairly based on genuine user engagement and effort.

Our summarized contributions are as follows:

- *Development of a Detection Method for M2E Fraud:* We present an AI-based detection method specifically designed to address the unique challenge of detecting fraudulent activities in Move-to-Earn (M2E) platforms. By distinguishing genuine human movements from those generated by automatic walking devices, our approach targets a critical and underexplored issue in the M2E ecosystem. Our method provides a robust solution for ensuring the integrity of M2E platforms. The effectiveness of our approach is demonstrated by achieving an F1-score of 0.997 on datasets containing auto-walker-generated movements and an F1-score of 1.000 on datasets of genuine human gait. To the best of our knowledge, this is the first method explicitly developed to protect M2E platforms from fraudulent behavior, thereby fostering trust and reliability in digital wellness ecosystems.
- *A Comparative Model Study:* We evaluate and compare the performance of several models, each selected for its potential to accurately discriminate genuine human gait data and those generated by auto-walkers. The models to be compared include a Convolutional Neural Network (CNN), which are designed to capture and analyze spatial and temporal features within the data. Beyond deep learning models, we include traditional machine learning models in our comparative study: Random Forest (RF), Support Vector Machine (SVM), and k-Nearest Neighbor (kNN). This comprehensive model comparison illuminates the most effective techniques for distinguishing between human and auto-walker data patterns.
- *Evaluation of the Auto-walker Detection Model in M2E Ecosystems:* We systematically evaluate the effectiveness of our proposed method specifically against the prevalent use of simplistic mechanical devices, commonly referred to as auto-walkers or automatic walking devices. These devices are designed to mimic human movements, thereby generating false gait data without physical activity on the part of the user. Our evaluation demonstrates the practical applicability of our model by distinguishing genuine physical activities from those generated by such devices.
- *Generalizability of Model Across Unseen Data:* Our method has exceptional generalizability, evidenced by its consistent accuracy in identifying problematic activity across both new, unseen gait data from different individuals and auto-walker data generated by various devices. We use a total of six open gait datasets and gait data from automatic walking devices measured with various smartphones. Under unbiased and transparent evaluation, our model has an F1-score of 0.994 on the unseen auto-walker datasets and an F1-score of 1.000 on the unseen genuine datasets. This robustness, tested across diverse datasets, showcases the model’s universal applicability and underscores its potential as a reliable tool in real-world scenarios, irrespective of the automatic walking device types and the smartphones.

## 2. Motivation

There is a burgeoning class of M2E applications that incentivize physical activity without necessitating high initial investments, such as the purchase of NFT sneakers. Examples of such applications include Charity Miles [10], Sweatcoin [11], Evidation [12], BetterPoints [13], and Runtopia [14]. These platforms provide a compelling proposition by rewarding users for engaging in physical activities and eliminating the barrier of costly digital assets. However, this ease of access makes these platforms particularly attractive to actors seeking to maximize rewards through minimal effort. Notably, devices such as automatic walking devices can artificially generate up to 10,000 steps per hour without any actual physical movement, presenting a significant challenge in distinguishing genuine walking activity from auto-walker activity.

The integrity of M2E platforms is crucial to their success. Auto-walker activity not only undermines trust in these platforms but also artificially inflates the distribution of rewards, thereby jeopardizing the economic model. Consequently, there is an urgent need to develop a sophisticated detection method capable of effectively countering such behavior. This need underpins the motivation for our research. It is imperative to safeguard the long-term viability of M2E applications by ensuring that rewards are distributed appropriately and that user trust and engagement are maintained. By addressing these challenges, we aim to reinforce the foundational principles upon which the M2E ecosystem is built, creating a secure environment, where users can participate in physical activities and legitimately earn rewards.

## 3. Related Works

**Human Activity Recognition and Gait Analysis.** Extensive research has been conducted in human activity recognition (HAR) and gait analysis, focusing on classifying various human movements such as walking, running, and sitting, as well as analyzing gait patterns for disease diagnosis [2,3,4,5,6,7,8]. Many studies have leveraged machine learning and deep learning models to recognize motion patterns or detect abnormalities in human gait.

For instance, Kaur et al. proposed a machine learning-based method to diagnose multiple sclerosis by analyzing spatiotemporal and kinematic gait features, demonstrating the potential of gait data in clinical applications [6]. Similarly, Takallou et al. developed a deep learning-based approach to diagnose peripheral arterial disease using accelerometer data collected from a wearable device placed on the waist, emphasizing the role of sensor-based motion analysis in disease detection [7]. In another study, Del et al. employed a statistical analysis-based method to diagnose Parkinson’s disease (PD) using accelerometer-based body-worn monitors, highlighting the effectiveness of accelerometer signals in identifying neurodegenerative disorders [8].

Beyond medical applications, several studies have explored HAR using various classification techniques. Raj et al. applied Convolutional Neural Networks (CNNs) to classify different human activities, demonstrating the capability of deep learning in recognizing motion patterns [2]. Nurwulan et al. utilized Random Forest models for HAR, while Chathuramali et al. proposed a Support Vector Machine (SVM)-based approach, and Mohsen et al. investigated the use of k-Nearest Neighbors (k-NN) for activity classification [3,4,5].

While these studies have significantly advanced HAR and gait analysis, they do not address the fundamental distinction between genuine human gait and artificially generated gait produced by mechanical devices. The existing research primarily focuses on recognizing human movements or diagnosing medical conditions but does not consider cases where motion sensor data are manipulated or artificially induced, such as in fraudulent activities within Move-to-Earn (M2E) applications.

**Fraud Detection in Digital Platforms.** In contrast to HAR studies, research on fraud detection in digital platforms has largely focused on identifying anomalies in financial transactions and behavioral patterns. Traditional fraud detection methods primarily rely on analyzing complex relational patterns within financial networks to flag suspicious transactions or activities [9]. These approaches have been effective in detecting fraudulent behaviors in financial systems but are not directly applicable to motion-based fraud detection, as they do not account for the sensor-driven manipulation of physical activities. In M2E applications, users are rewarded based on their physical activity data, making motion-based fraud a novel and emerging concern. Fraudsters can exploit low-cost auto-walkers (priced under USD 5) to artificially generate movement and accumulate rewards without engaging in actual physical activity. Despite the growing adoption of M2E platforms, there is currently no dedicated research addressing the detection of motion-based fraud or differentiating between genuine and artificially generated movement.

This critical research gap underscores the need for novel fraud detection techniques that leverage motion sensor data to ensure the integrity of M2E ecosystems. Unlike traditional fraud detection methods that primarily target financial transactions, effective fraud prevention in M2E applications requires the ability to distinguish genuine human gait from artificially generated movement.

## 4. Attack Model

We consider an actor who seeks to obtain a large reward in M2E ecosystems at a minimal cost: by using simplistic devices commonly referred to as auto-walkers or automatic walking devices. These devices can deceive activity tracking systems with simple, repetitive movements, effectively registering physical activity even though the user has not performed it. Such a strategy enables actors to gain rewards with minimal effort, bypassing the need for direct manipulation of the app’s software or its internal data and avoiding the costs associated with genuine physical activity. Accordingly, we do not consider actors who directly manipulate app software or internal data in this paper. In response to these challenges, our goal is to devise a robust model that detects such auto-walker activity facilitated by these simple devices.

## 5. Our Method

Our method is specifically designed to detect and distinguish automated walking patterns from genuine human gait data, ensuring the integrity of M2E platforms. Our approach not only identifies fraudulent walking patterns generated by auto-walkers but also accurately classifies genuine human movements as valid.

**Figure 1:**
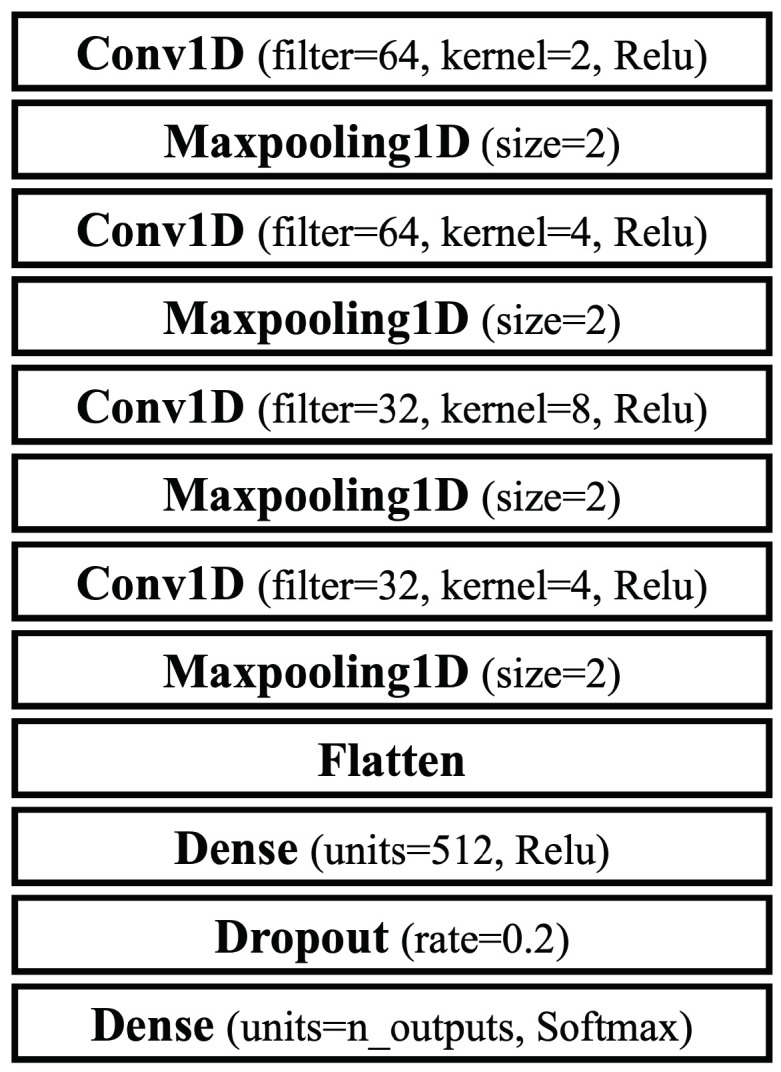
The layer configurations of a CNN model.

To provide a meaningful comparison, we evaluate and compare the performance of several widely used models in behavior analysis [2,3,4,5]. We integrate both deep learning and traditional machine learning techniques to perform a comprehensive evaluation of various model architectures. This includes models like Convolutional Neural Networks (CNNs), which are designed to capture and analyze spatial and temporal features within motion data. Additionally, beyond deep learning approaches, we include traditional machine learning models such as Random Forest (RF), Support Vector Machine (SVM), and k-Nearest Neighbors (kNN) in our comparative study. A brief description of the models used in our evaluation is provided below, while the detailed layer configurations of the deep learning models are illustrated in Figure 1.

- **CNN Model:** We evaluate a CNN model, which is particularly effective at capturing the spatial and temporal features of the data [15].
- **RF Model:** We evaluate a Random Forest model, which is a robust ensemble method that combines multiple decision trees to improve generalizability and reduce overfitting [16].
- **SVM Model:** We also evaluate a Support Vector Machine model, which works well with high-dimensional data and employs a kernel trick to transform data into higher dimensions [17].
- **kNN Model:** We finally evaluate a k-Nearest Neighbor model which is a simple, intuitive model that classifies data points based on the majority vote of their k-Nearest Neighbors [18].

To effectively distinguish genuine gait data from those of auto-walker movements, we implement a hybrid classification approach that combines deep learning models with binary SVM [19,20,21]. Initially, deep learning models are trained for multi-class classification to differentiate between N+1 classes, where N represents the types of auto-walker movements, and the additional class represents genuine gait data. Each deep learning model generates a probability vector indicating the likelihood of the input data belonging to each class. For final classification, these probability vectors are fed into a binary SVM model. The SVM is trained to label genuine gait data as legitimate (class 1) and auto-walker data as illegitimate (class 0), enabling the binary model to make the ultimate decision. This hybrid framework leverages the feature extraction capabilities of deep learning models while enhancing classification robustness with the binary SVM, ensuring the accurate differentiation between genuine and fraudulent movements.

We construct and evaluate the models in two ways according to model type. First, for deep learning models, since each model acts as a feature extractor for genuine and auto-walker data, we train each model to perform multi-class classification: If the auto-walkers have N movement types, the models are trained to classify N+1 multi-class classification problems, including the gait class (becoming class 0). Our ultimate goal is for the model to determine the genuine gait data as legitimate and auto-walker data as illegitimate data, which becomes a binary classification problem. Accordingly, we combine SVM models to deep learning models. Each deep learning model generates a probability vector indicating the likelihood of each data belonging to a particular class. Subsequently, the input data are classified based on the class corresponding to the highest probability element in the vector. These probability vectors are labeled as class 1 (i.e., True) for the genuine gait data and class 0 (i.e., False) for the auto-walker data, and then they are fed into the binary SVM model. The binary model makes the final classification decision regarding whether the data are genuine or not.

For the traditional machine learning models, we extract features from the data and then feed them into each model. Similarly, since our goal is to determine if the input data are genuine, we label those gait data (i.e., the extracted features from genuine gait data as class 1 and auto-walker data as class 0), and then we train each machine learning model.

**Feature Extraction.** To effectively distinguish between genuine human gait and the movements generated by automated devices, we develop a comprehensive feature engineering approach specifically tailored to the unique challenges of M2E fraud detection. By extracting meaningful features from raw gait data, we aim to capture the nuanced differences between human and mechanical movement patterns.

Our approach begins with the extraction of statistical features that represent the overall characteristics of raw gait signals, which are widely used in motion analysis [22]. These features include integrated absolute value, mean absolute value, variance, root mean square, standard deviation, mean value, and median absolute deviation. Additionally, we calculate simple moving averages (using simple, advanced, absolute simple, and absolute advanced methods), skewness, kurtosis, interquartile range, energy, and entropy (calculated using four different methods). These features provide a comprehensive statistical summary of the data, capturing global trends, variability, and temporal characteristics.

To improve the model’s ability to capture temporal patterns specific to gait data, we additionally extract peak-related features. These include the indices and heights of the three highest peaks in the raw gait signals. Furthermore, we calculate the differences in indices and heights between adjacent peaks, capturing the temporal and amplitude relationships within the gait cycle. For example, if the peak indices and heights are denoted as $\left(x_{1},y_{1}\right)$, $\left(x_{2},y_{2}\right)$, and $\left(x_{3},y_{3}\right)$, the derived features include $\left(x_{2}-x_{1},y_{2}-y_{1}\right)$, and $\left(x_{3}-x_{2},y_{3}-y_{2}\right)$. These features are particularly effective at capturing periodic and structural differences between human and mechanical movements. Since each gait dataset consists of six distinct signals (three axes from both accelerometer and gyroscope sensors), this feature extraction process results in a total of 174 features per dataset. By systematically incorporating both statistical summaries and temporal peak characteristics, this feature set is uniquely tailored to address the complexities of M2E fraud detection, providing a rich representation of the data that facilitates robust classification.

**Figure 2:**
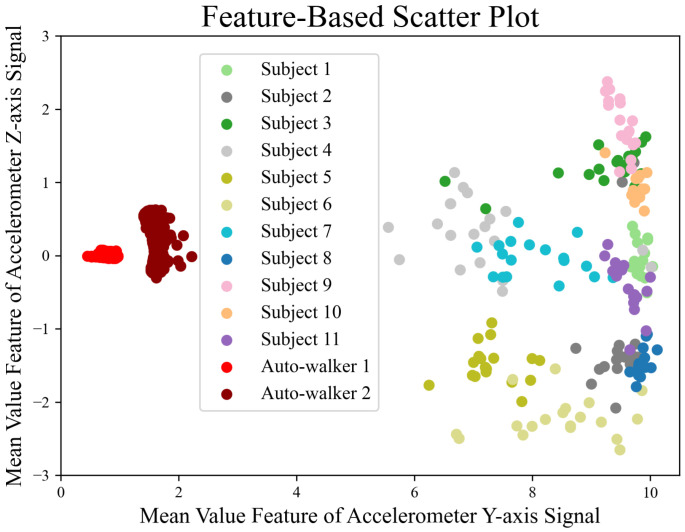
Scatter plot according to the features extracted from the data of 11 participants in the GeoTecINIT dataset and data of auto-walkers 1 and 2.

**Figure 3:**
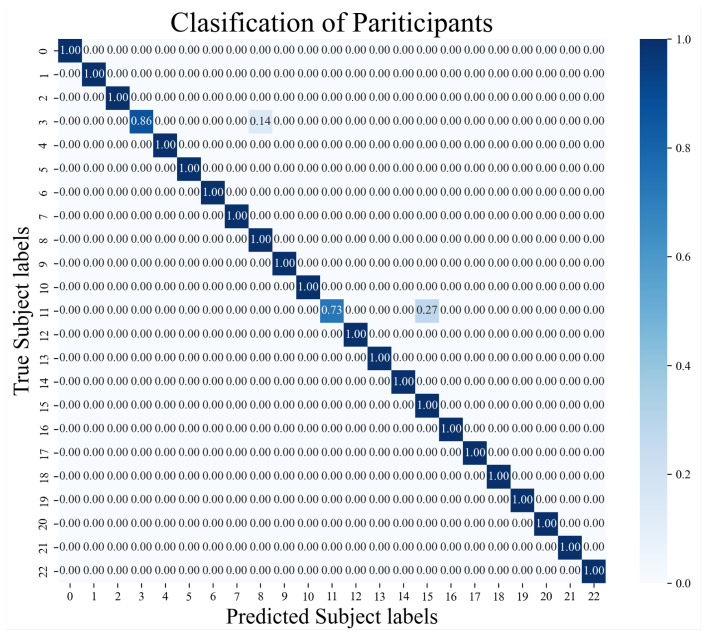
Results of classifying all 23 participants in the GeoTecINIT dataset using our features.

We evaluate whether our features are appropriate for gait data. To this end, we employ a scatter method because feature points typically cluster by participant if the features extracted from their gait data are associated. Conversely, a lack of association results in the features being dispersed. It is noted that at least two features are necessary to generate a scatter plot. However, given that the feature dimensions exceed two, we utilize a Random Forest model to classify all 23 participants in the GeoTecINIT dataset [23]. From the complete set of features, we then select two. The RF model provides a feature importance score, indicating the utility of each in classifying the participants. Figure 2 shows a scatter plot of the features extracted from the gait data of 11 participants in the GeoTecINIT dataset, alongside the data from auto-walkers 1 and 2. The clustering observed between features extracted from the 11 participants and those from the auto-walkers suggests that these features effectively capture the characteristics of the gait data. Among the total 174 features, the top 15 in terms of importance are listed in Table 1. Figure 3 shows the results of classifying all 23 participants in the GeoTecINIT dataset, indicating that our features well reflect the characteristics of each participant’s gait data.

## 6. Experimental Setup

### 6.1. Open Gait Datasets

To evaluate the performance of the model in identifying genuine human gait as legitimate, we utilize six publicly available gait datasets. These datasets contain extensive gait signals collected from multiple participants engaged in walking activities. The diverse activities recorded under real-world conditions provide a robust foundation for developing and testing the model. However, these datasets do not consistently provide detailed demographic information about participants, such as age, physical condition, or other personal attributes. To address this limitation, we rely on the inherent diversity within the datasets, which are collected in varied environments and contexts. This variability helps ensure a certain level of robustness in the data. Additionally, the use of publicly available datasets ensures an unbiased and transparent evaluation of the model. A summary of the open datasets is provided in Table 2.

**ADL Dataset.** The Activities of Daily Living (ADL) dataset [24] comprises data from the motions performed during five different daily activities: walking, running, standing, walking up the stairs, and walking down the stairs. Data were collected from a group of 25 participants in Portugal, consisting of 15 men and 10 women. The age range of the participants spanned 16 to 60 years old. For the data measurement, each participant was equipped with a BQ Aquaris 5.7 smartphone [25] placed in a waistband. The built-in accelerometer and gyroscope sensors measured data at a frequency of 100 Hz, and the magnetometer sampled at 50 Hz. The data measurements were performed in different environments, such as in a hall, on a street, and in other places. Since M2E apps generally count walking steps, only the walking data from this dataset were utilized. Additionally, we exclusively used data recorded by accelerometer and gyroscope sensors for our evaluation.

**FLAAP Dataset.** The FLAAP dataset [26] contains data from ten activities—including walking, sitting, standing, jogging, sitting cross-legged, lying down, walking in a circle, going up the stairs up, going down the stairs, and sitting up—measured from eight different participants. Participants were all men and had an average of 169.125 cm in height, 66.00 kg in weight, and 29.75 years in age. A smartphone was placed at the center of each participant’s body and the built-in accelerometer and gyroscope sensors measured data for each activity at a sampling frequency of 100 Hz. The data measurements were performed at Banaras Hindu University in India. Note that only data for walking and walking in a circle were used for our evaluation.

**GeoTecINIT Dataset.** The GeoTecINIT dataset [23,27] contains data pertaining to sequences of specific activities—such as sitting, standing up, walking, turning, and sitting down—measured from 23 participants. The participants represented diverse age demographics, ranging from 23 to 66 years old, and gender balance, featuring a male-to-female ratio of 56% to 44%. Data were measured using built-in sensors at a sampling rate of 100 Hz while the participants placed a Xiaomi Poco X3 Pro smartphone in their left pocket and wore a TicWatch Pro 3 GPS smartwatch on their left wrist. The data measurements were performed at Jaume I University in Spain. Note that only walking data were used for our evaluation.

**MotionSense Dataset.** The MotionSense dataset [28,29] comprises data from six different activities: going down the stairs, going up the stairs, walking, jogging, sitting, and standing. Data were collected from 24 participants, including 14 males and 10 females. The participants’ weight ranged from 40 kg to 100 kg, their height from 161 cm to 190 cm, and their age from 18 to 35 years. An iPhone 6s, located in the front pocket of the participant’s pants, measured data using built-in accelerometer and gyroscope sensors at a sampling rate of 50 Hz. The data measurements were performed around the Queen Mary University of London’s Mile End campus. Note that only walking data were used for our evaluation, and the data were upscaled to 100 Hz to match the sampling frequency of other datasets.

**Nazli Dataset.** The Nazli dataset [30] contains gait signals collected from 93 participants. The participants consisted of 47 males and 46 females, and their weight ranged from 44 kg to 137 kg, their height from 144 cm to 204 cm, and their age from 17 to 53 years. One smartphone was placed on the right thigh and one on the left side of the waist, and gait signals were measured using built-in accelerometer and gyroscope sensors while each participant walked between two endpoints (320 m). The dataset did not include details regarding the locations of the measurements or the sampling rate. Nonetheless, we inferred that the sampling rate was approximately 100 Hz by analyzing the timestamps in each data file.

**USC-HAD Dataset.** The USC-HAD dataset [31,32] contains data of twelve different activities: walking forward, walking to the left, walking to the right, walking up the stairs, walking down the stairs, running forward, jumping, sitting, standing, riding the elevator up, and riding the elevator down. Data were collected from 14 participants, including seven males and seven females. The participants’ weight ranged from 43 kg to 80 kg, their height from 160 cm to 185 cm, and their age from 21 to 49 years. A MotionNode [33] located in the participant’s front right hip pocket measured data at a sampling rate of 100 Hz utilizing its built-in accelerometer and gyroscope sensors. Data collection occurred across a number of days and was conducted in both indoor and outdoor settings. Note that only data for walking forward, walking to the left, and walking to the right were used for our evaluation.

### 6.2. Auto-Walking Devices

**Figure 4:**
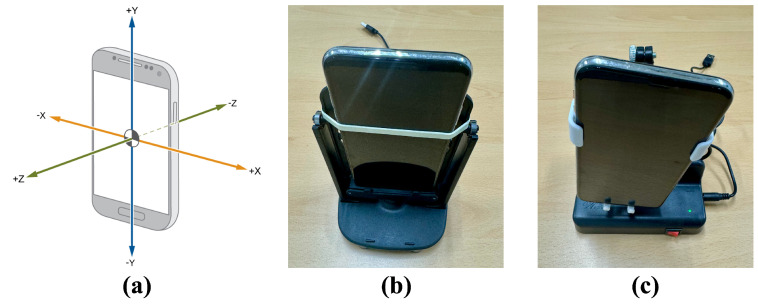
The orientation axes of a general smartphone and two types of auto-walkers: (**a**) X, Y, and Z orientation axes, (**b**) auto-walker 1 (rotating around X-axis), and (**c**) auto-walker 2 (rotating around the Z-axis).

To evaluate the performance of models in detecting data generated by auto-walking devices, we employ two types of auto-walkers (c.f., there are only two types on the market). These devices are distinguished by their rotational movements: One type rotates around the X-axis, while the other revolves around the Z-axis. We denote the device rotating around the X-axis as auto-walker 1, and the device rotating around the Z-axis as auto-walker 2. Figure 4 shows the orientation axes (X, Y, Z) of a standard smartphone and the two types of auto-walking devices. Each auto-walking device was purchased from AliExpress [34] at a low cost of approximately USD 1 and USD 5, respectively.

**Figure 5:**
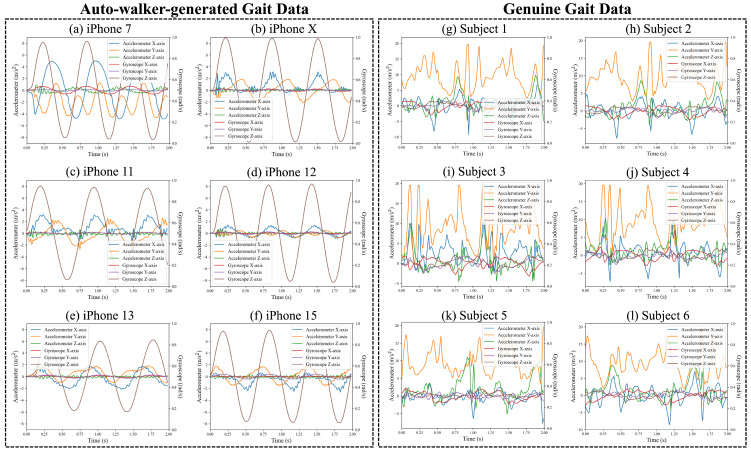
Comparison of auto-walker-generated and genuine gait data.

We utilize six smartphones, namely, the iPhone 7, X, 11, 12, 13, and 15. Figure 5a–f illustrate the accelerometer and gyroscope data measured from each smartphone using auto-walker 1, the device that rotates around the X-axis. Despite the identical movement of the auto-walker, each smartphone records distinct sensor data.

To provide a clearer comparison between auto-walker-generated and genuine human gait, we include accelerometer and gyroscope data from six randomly selected subjects from the ADL open dataset. These genuine gait signals are presented alongside the auto-walker data in Figure 5g–l, allowing for a direct comparison of motion patterns. The comparison highlights the distinct characteristics of natural human gait, which exhibits greater variability due to individual biomechanics, in contrast to the periodic and structured oscillations observed in auto-walker-generated movement. Although the difference between generated and genuine gait signals is visually apparent, M2E applications often fail to distinguish them and incorrectly classify artificially generated motion as real human activity. This misclassification occurs because human gait also inherently follows a periodic pattern, making it challenging to differentiate from simulated movement. This limitation further underscores the need for a robust classification model capable of capturing these differences to prevent fraudulent activity in M2E applications.

**Data Measurements.** Movement data from the two types of auto-walking devices were measured using different iPhone models. Data from auto-walker 1 were measured over a total of 3.08 h, while auto-walker 2 data were measured for a total of 1.17 h. Additionally, to evaluate the detection performance of the models over time, movement data were measured for auto-walkers 1 (for a total of 0.41 h) and auto-walker 2 (for a total of 0.42 h) using both the iPhone 7 and iPhone 13 after a period of two weeks. In total, 5.07 h of data were measured for the movements of auto-walkers 1 and 2.

## 7. Performance Metrics

In this section, we define the performance metrics used to evaluate our method. An important factor when evaluating these model is that both must identify genuine gait data as legitimate and gait data generated by the auto-walkers as illegitimate.

- **False Negative Rate (FNR)**: The rate of data generated by auto-walkers but determined to be genuine (i.e., the rate of false rejections).
- **False Positive Rate (FPR)**: The rate of genuine gait data but determined to be illegitimate (i.e., the rate of false acceptances).
- **Equal Error Rate (EER)**: The point at which FNR and FPR are equal, a point that is widely used to measure the performance of a model.
- **Precision**: The ratio of data correctly classified as belonging to a specific class out of all the data the model predicted to belong to that class, indicating that Precision focuses on reducing false positives.
- **Recall**: The ratio of data in a class that the model correctly classified out of all the data in that class, indicating that Recall, also known as sensitivity, focuses on reducing false negatives.
- **F1-score**: The harmonic mean of Precision and Recall, which is widely applied to balance Precision and Recall and is used when evaluating classification models. The F1-score is calculated as
  $$\begin{array}{c} 2\times \frac{\mathrm{Precision}\times \mathrm{Recall}}{\mathrm{Precision}+\mathrm{Recall}} \end{array}$$

## 8. Evaluation

In this section, we present how to divide datasets into a training dataset and a test dataset, how to train the models, and what the optimized hyperparameters of each model are. The performance of each model is evaluated based on whether it can determine genuine gait data as genuine and detect auto-walker data as illegitimate.

### 8.1. Data Configuration for Training and Testing Models

Our evaluation of the auto-walker detection model encompasses both seen and unseen data to comprehensively evaluate performance. In other words, other gait signals from participants included in the training set (i.e., other data from seen classes) are expected to be accurately classified as legitimate. Also, signals from other participants not included in the training set (i.e., data from unseen classes) should also be correctly identified as genuine data, affirming the model’s ability to generalize across different individuals. Similarly, data for the auto-walkers measured with a specific smartphone model included in the training set (i.e., other auto-walker data from seen classes) must be detected as illegitimate, and the data for the auto-walkers measured with other smartphone models not included in the training set (i.e., auto-walker data from unseen classes) must be also detected as illegitimate.

This subsection thus details how to configure data into training/test sets and additional test sets on unseen data to ensure a thorough evaluation of our model’s performance.

**Training/Test Data.** Initially, we preprocess the data from each dataset by segmenting them into arrays with dimensions of (200, 6), representing two seconds of data from a three-axis accelerometer and a three-axis gyroscope. To train and test our model, we utilize complete data from all participants in the FLAAP dataset (eight out of eight participants) and the GeoTecINIT dataset (23 out of 23 participants), along with a subset from the Nazli dataset (59 out of 93 participants). Each participant’s data are then split into training and test sets at a ratio of 0.8 to 0.2, respectively. As a result, within the FLAAP dataset, the total shapes for the training and test sets are (1168, 200, 6) and (290, 200, 6), respectively. For the GeoTecINIT dataset, these dimensions are (378, 200, 6) for training data and (70, 200, 6) for the test data. In the case of the Nazli dataset, the training and test sets are shaped as (11948, 200, 6) and (2928, 200, 6), correspondingly. We use data from the remaining 34 out of 93 participants in the Nazli dataset, which are not utilized in the training sets, to evaluate the model’s performance on unseen gait data. Additionally, we train and test our model using gait data generated by auto-walkers 1 and 2 measured with the iPhone 7 and 13. The training set includes data from auto-walkers 1 and 2 measured with iPhone 7 and 13, totaling 2.11 h and 0.59 h, respectively. For the test set, the data for auto-walkers 1 and 2 are measured for a total of 0.53 h and 0.15 h, respectively.

In summary, the training and test sets for the seen gait data have shapes of (13494, 200, 6) and (3288, 200, 6), respectively, sourced from a total of 90 participants. The shapes of the training and test sets for the seen auto-walker data are (4857, 200, 6) and (1213, 200, 6), respectively.

**Unseen Test Data.** For unseen gait data, we use data from participants not included in the training sets of the open datasets. Specifically, the ADL dataset provides gait signals from 25 participants, resulting in a test set shaped as (1393, 200, 6). The MotionSense dataset contributes gait signals from 24 participants, with a dataset shape of (3336, 200, 6). From the Nazli dataset, we include the gait signals of the remaining 34 participants, leading to a shape of (7852, 200, 6). Lastly, the USC-HAD dataset offers data from 14 participants, with a dataset shape of (6295, 200, 6). As for the unseen auto-walker data, data from auto-walkers 1 and 2 were measured with the iPhone X, 11, 12, and 15 models.

Therefore, the combined test sets for the unseen gait data have a total shape of (18876, 200, 6), sourced from a total of 97 participants, and the shape of the test sets for the unseen auto-walker data is (1574, 200, 6)

### 8.2. Model Training and Hyperparameter Optimization

**Deep Learning Models.** As mentioned in Section 5, we employ a deep learning model, CNN. Initially, for multi-class classification, we set class labels as follows: gait data are labeled as class 0, data for auto-walker 1 as class 1, and data for auto-walker 2 as class 2. Each model is trained using the ‘categorical_crossentropy’ loss function and the Adaptive Moment Estimation (Adam) function, with parameters set to a batch size of 40, 10 training steps, and 10 epochs.

For our ultimate goal, which is to determine the gait data or auto-walker data, we combine binary SVM models to the deep learning models. To train and optimize each SVM model, we employ the five-fold cross-validation method along with the ‘gridsearch’ function to tune its hyperparameters. These hyperparameters include kernels, regularization parameters, and kernel coefficients, which are chosen from predefined sets [‘linear’, ‘rbf’, ‘poly’], [0.1, 1, 10, 100], and [1, 0.1, 0.01, 0.001], respectively. Through this tuning process, the optimal hyperparameters for the CNN-SVM combination are found to be {‘kernel’: ‘poly’, *C*: 1, γ: 0.1}, as the polynomial kernel demonstrates superior performance in capturing non-linear gait variations compared to linear and RBF kernels. The selected *C* and γ values balance model complexity and classification performance, ensuring robustness across datasets.

**Machine Learning Models.** We evaluate three traditional machine learning models: a binary kNN model, a binary RF model, and a binary SVM model. Each model undergoes hyperparameter optimization via grid search to maximize classification accuracy.

For the kNN model, we tune the number of neighbors and weighting function, selecting from predefined sets [3, 5, 7, 9, 11] and [‘uniform’, ‘distance’], respectively. The best configuration is {‘n_neighbors’: 3, ‘weights’: ‘uniform’}, as a lower neighbor count preserves fine-grained gait distinctions, while larger values lead to excessive smoothing, reducing classification performance. For the RF model, the optimized hyperparameters are {‘n_estimators’: 200, ‘max_features’: ‘sqrt’, ‘max_depth’: 7, ‘criterion’: ‘gini’}. Using 200 estimators ensures classification stability, while ‘sqrt’ for ‘max_features’ improves generalization. A maximum depth of 7 prevents overfitting, and the ‘gini’ criterion provides better robustness across different sensor devices. For the SVM model, the best-performing hyperparameters are {‘kernel’: ‘poly’, *C*: 0.1, γ: 1}. Similar to the CNN-SVM model, the polynomial kernel outperforms linear and RBF kernels in capturing non-linear gait variations. A lower *C* value (0.1) prevents overfitting to device-specific noise, while a higher γ value (1) allows for more flexible decision boundaries, improving classification across datasets.

These optimized hyperparameters provide the best trade-off between classification accuracy and computational efficiency, ensuring reliable distinction between real and artificially generated gaits.

### 8.3. Evaluation on Auto-Walker Detection Models

**Figure 6:**
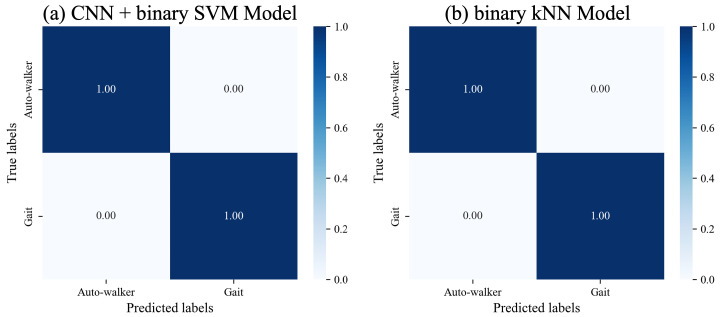
Classification results of seen gait data and seen auto-walker data for two selected models.

We use a dataset consisting of 4501 signal data, encompassing both seen gait data and data for auto-walkers 1 and 2, for evaluation purposes. The CNN model combined with binary SVM successfully distinguishes gait data generated by the auto-walkers from genuine gait data. Likewise, the machine learning models for binary classification, which are kNN, RF, and SVM models, distinguish to a high degree of accuracy between auto-walker data and genuine data. Figure 6a,b show the results of classifying seen gait data and seen auto-walkers data for the CNN model combined with SVM, and the binary kNN model. In the diagonal components, all values are given 1. This result implies that models are able to distinguish genuine gait data and auto-walker data with low error rates.

**Figure 7:**
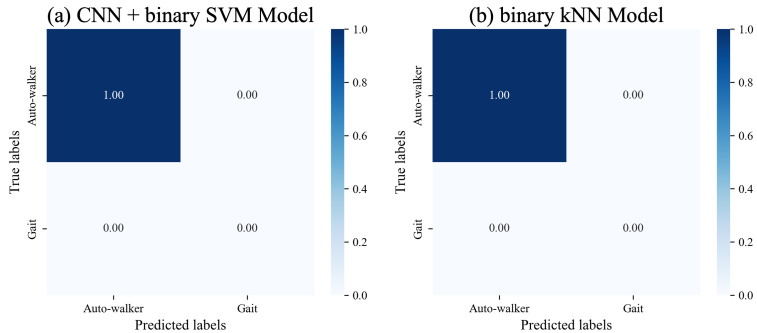
Classification results of auto-walker data after two weeks for two selected models.

**Evaluation on Model Performance over Time.** Furthermore, we evaluate the performance of each model to maintain accurate auto-walker data detection over time. After a two-week interval, we measure a total of 423 data for auto-walker 1 using the iPhone 7, along with 314 auto-walker data using the iPhone 13. Similarly, for auto-walker 2, 380 data were measured with the iPhone 7, and 369 data with the iPhone 13. The CNN model combined with SVM successfully detects auto-walker data measured after two weeks. Likewise, the machine learning models for binary classification, which are kNN, RF, and SVM, detect the illegitimate data. Figure 7a,b show the results of classifying auto-walker data after two weeks for the CNN model combined with the SVM, and the binary kNN model. In the first diagonal components, all values are given 1. This result implies that the models are able to detect auto-walker data accurately even as time elapses.

### 8.4. Evaluation on Model Generalizability Across Unseen Data

We evaluate the model’s ability to distinguish between genuine and fraudulent gait data under two specific unseen conditions. First, we test the model on gait data from users who were not included in the training dataset to assess its capacity to identify genuine gait patterns from previously unencountered individuals. Second, we evaluate the model using auto-walker data collected from smartphone models that are not part of the training data. Since identical movements can produce differing sensor readings depending on the smartphone model, this ensures that the model can generalize across device-specific variations. These evaluations demonstrate the model’s robustness and universal applicability in real-world M2E scenarios, where both new users and diverse devices are prevalent.

**Figure 8:**
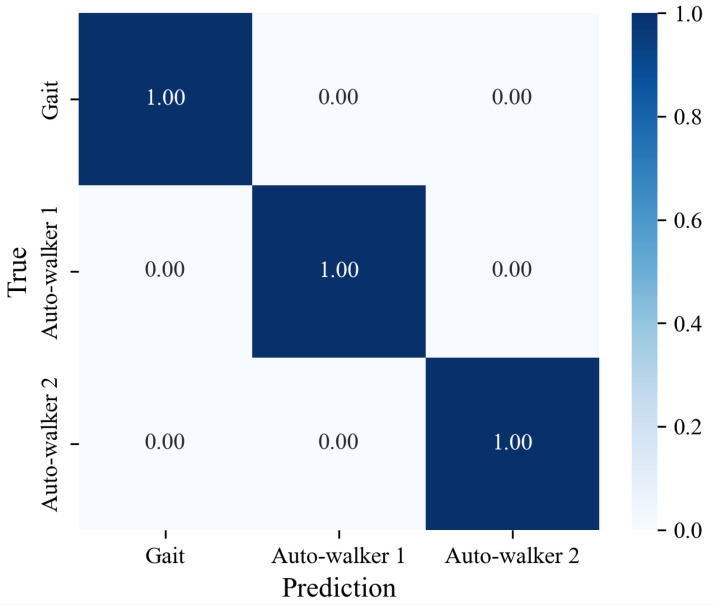
Classification results of unseen gait data, unseen auto-walker 1 data, and unseen auto-walker 2 data by a CNN model.

Since we use the CNN model as the feature extractor, we first check whether the model satisfactorily extracts features from data of the genuine gait dataset and the auto-walker datasets (i.e., whether it classifies them well). Figure 8 shows the result of classifying three unseen datasets (i.e., genuine gait dataset, auto-walker 1 dataset, and auto-walker 2 dataset). All diagonal components are 1.0. This implies that the CNN model distinguishes to a high degree of accuracy between the genuine gait datasets and the auto-walker datasets, even if the data are unseen.

**Figure 9:**
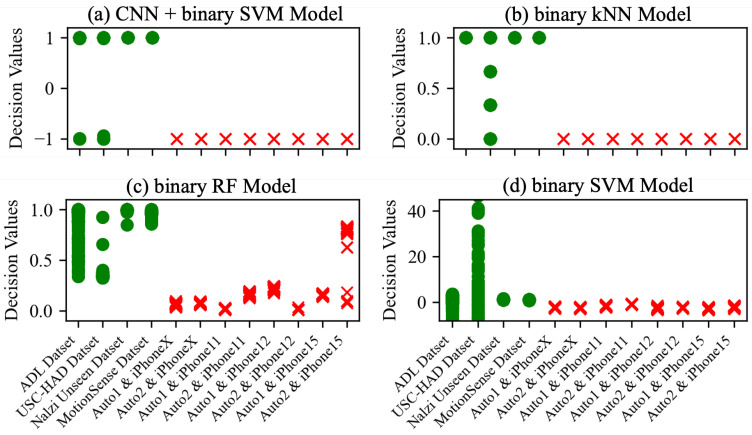
Decision values for each unseen dataset by model.

**Decision Values for Unseen Gait Data by Model.** After extracting features from the data, whether using deep learning models or those specified in Section 5, our goal is to ultimately determine genuine gait data as legitimate and auto-walker data as illegitimate. We analyze decision values for each unseen dataset that indicates in which class unseen data are classified when they are given to the model. The ‘decision_function’ obtains the decision values for the SVM model, and ‘predict_proba’ for the kNN and RF models. The ‘decision_function’ of the SVM model provides a raw decision score for each data point, which is a value that indicates how far the data are from the decision boundary (i.e., which class they belong to). On the other side, the ‘predict_proba’ estimates the probability that the data belong to each class. The kNN model finds the k-Nearest Neighbors for given data, and count the number of neighbors belonging to each class among these neighbors. The probability of belonging to a class is then calculated by dividing the number of neighbors in each class by the total number of neighbors (k). For the RF model, the probability that the given data belong to each class is calculated independently from all trees, and the final class probability is calculated as the proportion of trees that selected that class out of all trees. Accordingly, for the binary classification, the output of the ‘predict_proba’ is a vector with two elements (i.e., the probability value of each class), and the sum of each element is 1.0. We consider the second element value of the probability vector as the decision value. Figure 9 shows the decision values for each unseen dataset derived from all models. The decision values of the data in the Nazli unseen dataset and the MotionSense dataset are distributed around 1.00, but the decision values of the ADL dataset and USC-HAD dataset have a wide distribution. Table 3 illustrates the minimum and maximum of the decision values for each unseen gait dataset by model.

**Decision Values for Unseen Auto-walker Data by Model.** Similar to the unseen gait datasets, we analyze the decision values of models for each unseen auto-walker dataset. Figure 9 shows all the decision values for each unseen auto-walker datasets derived from all models. For all deep learning models, the decision values of the data in the all unseen auto-walker datasets are distributed around −1.00. The number of unseen gait data with a decision value of −0.99 or less for each model is as follows: For the CNN model 1 combined with the SVM model, there are four in the ADL dataset and 183 in the USC-HAD dataset, while for the CNN model 2 combined with the SVM model, there are five in the ADL dataset and 12 in USC-HAD dataset. For the CNN-LSTM model combined with the SVM model, there are 14 in the ADL dataset and 5448 in the USC-HAD dataset, and for the CNN-GRU model combined with the SVM model, there are 113 in the ADL dataset and 5890 in the USC-HAD dataset. The decision values of all unseen autor-walker datasets are 0.00 for the binary kNN model, and the number of unseen gait data with a decision value of 0.01 or less is 5688 in the USC-HAD dataset. For the binary RF model, the decision values of all unseen auto-walker datasets range between 0.005 (from auto-walker 1 and iPhone 11) and 0.835 (from auto-walker 2 and iPhone15), and the number of unseen gait data with a decision value of 0.84 or less is 85 in the ADL dataset and 6293 in the USC-HAD dataset. Last is the the binary SVM model, for which the decision values of all unseen auto-walker datasets are distributed from −3.42 to −0.54 and the number of unseen gait data with a decision value of 0 or less is 93 in the ADL dataset and 6245 in the USC-HAD dataset. Even if we simply compare the range values of decision values, we can observe a significant overlap between the unseen gait datasets and the unseen auto-walker datasets, except for the CNN models combined with the SVM models.

**Overall FNR and FPR of All Unseen Data by Model.** To assess the overall performance of each model, we evaluate the False Positive Rate (FPR) and False Negative Rate (FNR) across all unseen datasets as a function of the threshold. Since each model generates decision scores on different scales, the optimal threshold range varies. The CNN + binary SVM model exhibits a threshold range of [−2, 2], as the CNN extracts refined, lower-dimensional features that result in a more compact and concentrated decision score distribution. In contrast, when using binary SVM alone, the input feature space remains higher-dimensional and more diverse, causing the decision scores to be more widely distributed and requiring a broader threshold range of [−4, 4]. Meanwhile, binary kNN and binary RF output probability-based classification scores, resulting in a threshold range of [−0.25, 1.5]. These differences in threshold distributions reflect how each model processes feature variability and classification confidence.

**Figure 10:**
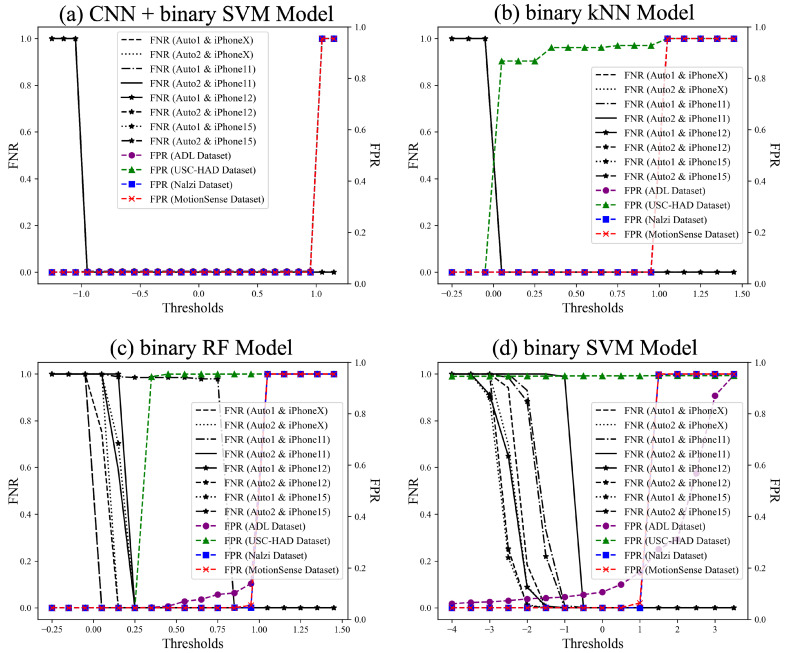
FPR and FNR of each unseen dataset by model.

Figure 10 presents the FPR and FNR of all unseen datasets based on the threshold for each model. Traditional machine learning models achieve high classification accuracy on seen test datasets and some unseen datasets but struggle with the USC-HAD dataset. In contrast, the CNN model, particularly when combined with SVM, exhibits consistently strong performance across all datasets. Although feature extraction is performed effectively, as discussed in Section 5, the lower performance of traditional models on the USC-HAD dataset can be attributed to its greater diversity in gait types. Models such as SVM, kNN, and RF rely on manually extracted features, making them less adaptable to highly variable gait patterns, which likely result in reduced classification accuracy. Conversely, the CNN model demonstrates superior feature extraction capabilities, automatically learning spatial and temporal representations from motion sensor data. This ability allows it to capture subtle variations in gait dynamics and generalize more effectively across diverse walking styles. As a result, the CNN model significantly outperforms traditional approaches on the USC-HAD dataset, demonstrating its robustness in handling datasets with high intra-class variability. This adaptability makes CNN more effective in distinguishing genuine gait from auto-walker motion across a wide range of unseen datasets. For better visual clarity, Figure 10 presents the overall trends, while detailed individual plots for each model are provided in Appendix A to enhance interpretability.

**Figure 11:**
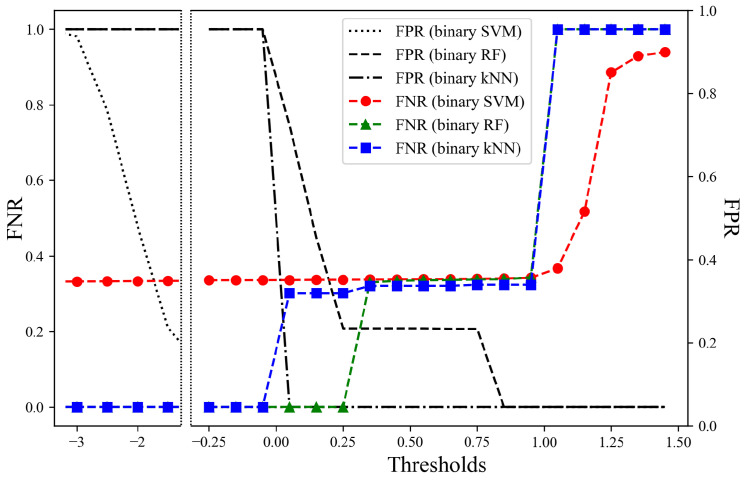
Overall FPR and FNR of total unseen datasets by machine learning model.

To evaluate the overall performance of each model, we obtain the FNR and FPR on all unseen datasets. The CNN model with the SVM has an EER of 0.001 based on a threshold of −0.990. In addition, Figure 11 shows the overall FNR and FPR for the machine learning models: the binary kNN model has an EER of 0.243 based on a threshold of 0.379, the binary RF model has an EER of 0.208 based on a threshold of 0.313, and the binary SVM model has an EER of 0.333 based on a threshold of −1.729. As a result, our model, which is a combination of the CNN model and the binary SVM model, has the lowest EER of 0.001 for all unseen datasets. This implies that it can distinguish between genuine gait data and auto-walker data even for data collected from participants or devices not encountered during model training.

### 8.5. Comprehensive Analysis of Model Performance in Detecting Auto-Walker Activity

Each model ultimately determines which class the data belong to based on their decision value. For the SVM model, if the decision value of the data is positive, they belong to the positive class (i.e., gait class); otherwise, they belong to the negative class (i.e, auto-walker class). The RF and kNN models output a probability vector with two elements (i.e., the probability value of each class) indicating which class the data belong to, and classify them into the class at the position of the largest element in the vector. Table 4 illustrates the Precision, Recall, and F1-scores for all seen and unseen datasets by model. In the case of auto-walker data, the Precision of all models except the CNN model combined with the SVM model is significantly lower than the Recall. A low Precision value indicates that among the results the model predicts as auto-walker data, there are more genuine gait data than auto-walker data. This means that the model does not learn the difference between auto-walker data and genuine gait data well. Additionally, in the case of genuine gait data, the Recall of all models except the CNN model combined with the SVM model is somewhat lower than Precision. A low Recall value means that the model predicts only some of the data that are actually genuine gait data as genuine gait data. In other words, this indicates that genuine gait data are not accurately identified and the completeness of the model is low.

## 9. Discussion

**Dataset Limitations and Generalizability.** This study evaluated the proposed method using six publicly available gait datasets comprising data collected from 187 participants. Additionally, we collected auto-walker data using iPhone 7, 13, X, 11, 12, and 15 to assess the model’s performance. These diverse datasets underscore the robustness of the model; however, several limitations remain. First, class imbalance in some datasets could bias the model’s predictions. For instance, genuine gait data are more prevalent than auto-walker data, potentially leading to a learning bias favoring the majority class. Future research could address this issue by exploring advanced techniques such as data augmentation or cost-sensitive learning. Second, variations in sensor placement, sampling rates, and recording conditions could result in inconsistencies in data distributions. These environmental and device-specific variations present challenges to the model’s generalizability. To address this, we divided the data into training, testing, and unseen datasets. Specifically, data from 90 participants were used for training and testing, while data from the remaining 97 participants were reserved exclusively for testing the model’s generalization capability. Additionally, auto-walker data collected from iPhone 7 and 13 were used for both training and testing, while data collected from iPhone X, 11, 12, and 15 were used solely for testing. This cross-device evaluation played a crucial role in assessing the model’s adaptability to unseen devices.

These experimental conditions are critical for evaluating the model’s performance and applicability in real-world scenarios. While class imbalance and data diversity remain limitations, the results demonstrate the model’s effectiveness across diverse scenarios, highlighting its potential for reliable fraud detection in M2E platforms.

**Participant Diversity.** One limitation of this study is the absence of detailed demographic information about the participants in the publicly available datasets used for evaluation. Specific factors such as age, physical condition, or other personal attributes are not consistently provided, which limits the ability to comprehensively analyze the model’s applicability across diverse population groups. To mitigate this limitation, we relied on the inherent diversity within the datasets, which were collected in varied environments and contexts. While this provides some degree of variability, it may not fully represent the wide range of potential users in real-world applications. Future research should prioritize datasets that include detailed demographic information to better evaluate the model’s performance and applicability across different user groups.

**Other Movement Patterns.** This study focuses on distinguishing genuine walking patterns from auto-walker movements to address fraud detection in M2E applications. Future work could expand the scope by incorporating additional movement patterns, such as running or cycling, to enable multi-class classification. This would enhance the model’s applicability to broader activity recognition tasks beyond fraud detection. Furthermore, analyzing walking patterns in the context of more diverse activities, such as distinguishing walking from sitting or jumping, could improve the model’s generalizability in real-world scenarios. Such extensions would make the system more robust and versatile for various applications.

**Use of More Complex Automatic Walking Devices.** We propose a model capable of detecting data generated by existing automatic walking devices that move in two patterns. Additional automatic walking devices with different types of movements may also be developed in the future. Our model demonstrates generalizability across various unseen datasets, and therefore, we expect that it will be able to identify auto-walker movements even in the face of more complex devices. Furthermore, such advanced devices will surely come at a higher price, potentially raising the cost for actors to engage in problematic activities.

**Comparison with Existing Methods.** Our study focuses on motion-based fraud detection in M2E applications, and to the best of our knowledge, no existing methods specifically address this issue. While traditional fraud detection techniques often target financial anomalies, they do not account for motion manipulation via mechanical devices. Consequently, we evaluated and compared the performance of several widely used models in anomaly detection and behavioral analysis. These models serve as reasonable baselines given their effectiveness in related domains and allow us to demonstrate the advantages of our proposed approach.

**Potential Application Scenarios.** Our study focuses on detecting fraudulent activities in Move-to-Earn (M2E) applications. Our method distinguishes genuine human movement from artificially generated motion using only motion sensor data (accelerometer and gyroscope), which is readily available in most smartphones and wearable devices. This enables seamless integration into existing M2E frameworks while also offering the potential for expansion into other domains. The ability to differentiate natural human gait from synthetic or artificially induced movements has broad applications in security, healthcare, and fair competition in motion-based systems.

One key application is wearable device security, particularly in fitness trackers and smartwatches. Many reward-based fitness apps can be exploited by artificially generating motion (e.g., attaching devices to oscillating objects). Our AI-based model enhances fraud detection, ensuring fair use and system integrity. In healthcare, our method aids physical rehabilitation monitoring by distinguishing genuine patient movements from assisted/artificial movements. This would improve the effectiveness of remote rehabilitation programs and ensure that patients receive appropriate feedback. Additionally, our model helps prevent cheating in exergames and virtual sports, where some users exploit automated movement devices for unfair advantages. Integrating our detection model ensures fair competition and gameplay integrity.

By extending our AI-driven motion fraud detection methodology to these areas, we believe that our approach has the potential to contribute beyond the M2E ecosystem, addressing security concerns, enhancing healthcare applications, and maintaining fairness in motion-based digital environments.

## 10. Conclusions

As M2E applications gain popularity, the misuse of automatic walking devices to simulate physical activity and unfairly claim rewards has emerged as a critical issue. To address this, we proposed an AI-based method capable of distinguishing genuine human movements from those generated by automatic walking devices. Utilizing six open gait datasets and auto-walker datasets measured across various smartphones, we evaluated the generalizability of our model. By optimizing the model configuration through comparisons with both deep learning and traditional machine learning approaches, our method achieved F1-scores of 0.997 on auto-walker datasets and 1.000 on genuine datasets, demonstrating its robustness and effectiveness. Our system ensures that rewards are fairly allocated based on genuine user engagement, enhancing the integrity and trustworthiness of M2E platforms. By tackling the challenges posed by auto-walker activity, our work contributes to building a more secure and equitable ecosystem for users. This ultimately supports the broader mission of promoting physical wellness through technology by ensuring that rewards reflect genuine effort and participation.

## Figures and Tables

**Table 1:** Top 15 features by importance.

Importance	Feature Description
0.022192	Mean value of accelerometer y-axis signal
0.021644	Advanced SMA of accelerometer y-axis signal
0.020901	Advanced SMA of accelerometer z-axis signal
0.020395	Height of the first peak in accelerometer y-axis signal
0.018841	Height of the third peak in accelerometer y-axis signal
0.017451	First difference of indices in accelerometer y-axis signal
0.017286	Simple SMA of accelerometer y-axis signal
0.016541	Simple SMA of accelerometer x-axis signal
0.016482	Absolute-advanced SMA of gyroscope y-axis signal
0.016093	Absolute-simple SMA of gyroscope y-axis signal
0.016029	Root mean square of gyroscope y-axis signal
0.015632	Energy of gyroscope z-axis signal
0.015478	Index of the first peak in accelerometer y-axis signal
0.015299	Mean value of accelerometer z-axis signal
0.014955	Root mean square of gyroscope z-axis signal

**Table 2:** Lists of open datasets used in our evaluation.

Dataset	#1	#2	#3	#4	#5	#6
	ADL	FLAAP	GeoTec	M.S.	Nazli	USC-HAD
Subjects	25	8	23	24	93	14
#(data) *	1393	1458	448	3336	22,728	6295
Freq.	100 Hz	100 Hz	100 Hz	50 Hz	100 Hz	100 Hz

* The number of datasets when one data shape is (200, 6).

**Table 3:** Range of decision values for each unseen gait dataset.

	ADL		USC-HAD		Nazli		MotionSense	
	Min	Max	Min	Max	Min	Max	Min	Max
CNN *	−1.00	1.00	−1.00	1.00	around 1.00		around 1.00	
bin. kNN	1.00		0.00	1.00	1.00		1.00	
bin. RF	0.34	1.00	0.32	0.93	0.85	1.00	0.86	1.00
bin. SVM	−21.38	3.71	$-1.68\times 10^{5}$	107.15	1.00	1.65	0.85	1.30

* Combined with the binary SVM model.

**Table 4:** Precision, Recall, and F1-scores for all datasets by model.

	Auto-Walker Data			Genuine Gait Data		
	Precision	Recall	F1-Score	Precision	Recall	F1-Score
CNN *	0.994	1.000	0.997	1.000	0.999	1.000
bin. kNN	0.315	1.000	0.480	1.000	0.727	0.842
bin. RF	0.280	0.883	0.426	0.980	0.715	0.827
bin. SVM	0.305	1.000	0.468	1.000	0.714	0.833

* Combined with the binary SVM model.

**Figure A1:**
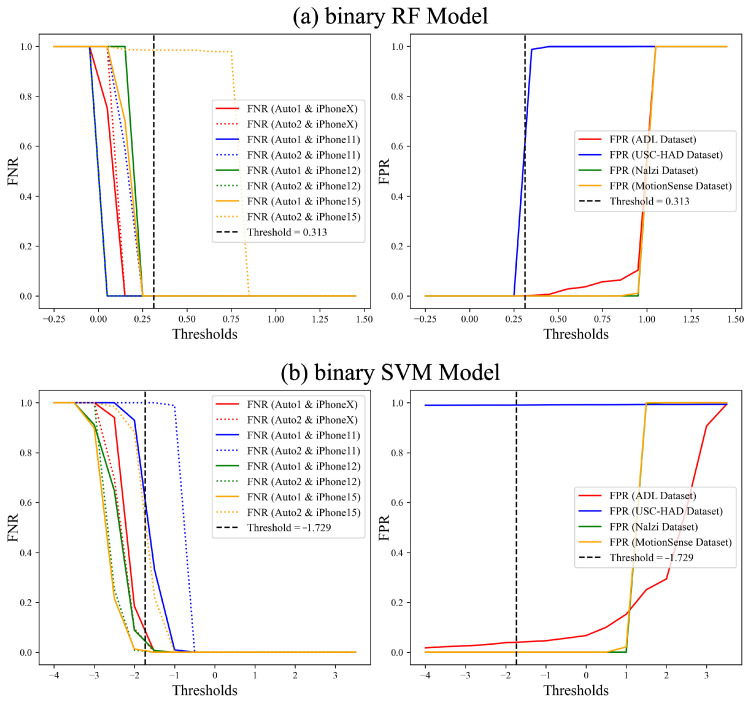
Individual FNR and FPR for each model and dataset.

## Data Availability

The data presented in this study are available at the following sources: ADL Dataset: https://data.mendeley.com/datasets/xknhpz5t96/2, FLAAP Dataset: https://data.mendeley.com/datasets/bdng756rgw/1, GeoTecINIT Dataset: https://zenodo.org/records/8398688, MotionSense Dataset: https://github.com/mmalekzadeh/motion-sense, Nazli Dataset: https://figshare.com/articles/dataset/Gait_Database/20346852, USC-HAD Dataset: https://sipi.usc.edu/had/, all accessed on 4 February 2025.
